# Changing the size of dendritic spines

**DOI:** 10.7554/eLife.91566

**Published:** 2023-09-07

**Authors:** Takeo Saneyoshi

**Affiliations:** 1 Department of Pharmacology, Kyoto University Graduate School of Medicine Kyoto Japan

**Keywords:** CaMKII, actinin, long-term potentiation, synapse, dendritic spine, protein kinase, Rat

## Abstract

Interactions between an enzyme kinase, an ion channel and cytoskeletal proteins maintain the structure of synapses involved in memory formation.

**Related research article** Curtis AJ, Zhu J, Penny CJ, Gold MG. 2023. Molecular basis of interactions between CaMKII and α-actinin-2 that underlie dendritic spine enlargement. *eLife* **12**:e85008. doi: 10.7554/eLife.85008.

Neurons connect to one another at sites called synapses, which are essential for creating and storing memories. Synapses are also dynamic and can undergo structural changes, such as increasing in size, that modify their strength. Structures called dendritic spines – which protrude from the dendrites that extend from the cell body of a neuron – have an important role in synapses as they contain an actin cytoskeleton that allows them to change shape and size.

An enzyme called CaMKII (which is short for Ca^2+^/calmodulin-dependent protein kinase II) also has a central role in the functional and structural changes that take place in dendritic spines during memory formation. During synapse strengthening, an influx of calcium ions through a receptor known as NMDAR, triggers the activation of CaMKII: the calcium ions form a complex with a messenger protein called calmodulin, and this complex binds to the regulatory segment of CaMKII. This influences the dynamics of the actin cytoskeleton and hence determines the size of the synapses. However, the details of this process – especially the link between CaMKII and the actin cytoskeleton at the molecular level – are not fully understood.

One candidate for linking CaMKII to the actin cytoskeleton is alpha-actinin-2, a structural protein that crosslinks actin filaments (Walikonis et al., 2001). This protein can anchor and organize other proteins in the dendritic spine, such as CaMKII (Robison et al., 2005), the receptor NMDAR (Wyszynski et al., 1997), and certain channel proteins that allow calcium ions to enter cells (Hall et al., 2013). Now, in eLife, Matthew Gold from University College London and colleagues – including Ashton Curtis and Jian Zhu as joint first authors – report new insights into the molecular interactions between CaMKII, alpha-actinin-2, and a subunit of NMDAR called GluN2B (Curtis et al., 2023; Figure 1).

**Figure 1.**
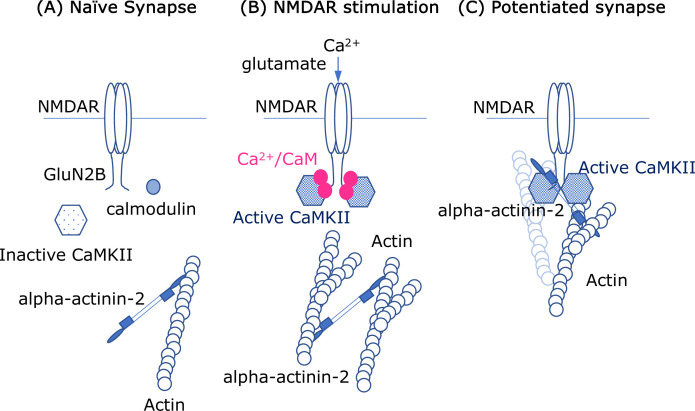
Molecular interactions and synapses. (**A**) In the naïve synapse, the only protein interactions are between actin filaments (blue) and alpha-actinin-2, a structural protein that crosslinks actin filaments. In particular, the enzyme CaMKII (hexagon shape) is inactive, and a messenger protein called calmodulin (CaM) does not interact with anything else. (**B**) When the NMDAR receptor in the cell membrane is activated by the neurotransmitter glutamate, calcium ions (Ca^2+^; solid pink circles) enter the cell. The ions form a complex with CaM, and this complex activates the enzyme CaMKII, which subsequently interacts with the GluN2B subunits in the intracellular region of the NMDAR receptor. Crosslinking of the actin filaments also causes the dendritic spine to increase in size. (**C**) In the enlarged synapse, the interaction between GluN2B, CaMKII and alpha-actinin-2 continues, even in the absence of Ca^2+^/CaM, and the enzymatic activity of CaMKII is maintained by binding to GluN2B. The complex formed by GluN2B, CaMKII and alpha-actinin-2 also associates with actin filaments to maintain the synaptic structure.

Using a proximity labeling assay method – which enables molecular interactions to be detected in situ – the researchers showed that CaMKII interacts with alpha-actinin-2 when the NMDAR receptor is stimulated, leading to an enlargement of the dendritic spine. The interaction takes place at a domain in alpha-actinin-2 called EF hands 1–4, which is known to bind calcium ions. Furthermore, when this domain was overexpressed, stimulation of the NMDAR receptor did not result in dendritic spine enlargement, suggesting that interaction via the EF1-4 domain has a critical role in structural changes to the dendritic spine.

Affinity and structural experiments showed that alpha-actinin-2 binds to the CaMKII enzyme at the same regulatory domain segment as calmodulin (which also contains EF hands) does. However, Curtis et al. found that the EF hands of alpha-actinin-2 clashed with one of the domains in the enzyme. Consistent with this, the inactive form of the enzyme cannot bind to alpha-actinin-2 via its regulatory domain segment. However, the interaction between the CaMKII enzyme and alpha-actinin-2 in the dendritic spine increased four hours after stimulation of the NMDAR receptor: this led Curtis et al. to hypothesize that, following stimulation, the configuration of the enzyme changes when it interacts with a subunit of the NMDAR receptor called GluN2B, and these changes enable alpha-actinin-2 to fully access the enzyme.

A pull-down assay – which detects interactions between proteins – revealed that GluN2B enhances the interaction between the CaMKII enzyme and alpha-actinin-2, independently of the concentration of calcium ions (Ca^2+^). This indicates that GluN2B enhances the binding of CaMKII with alpha-actinin-2 even in the presence of Ca^2+^/calmodulin, although the EF hands of alpha-actinin-2 have a lower affinity for CaMKII than the EF hands of Ca^2+^/calmodulin. This provides important insights into a reorganization process for the molecular architecture of synapses.

Taken together, the findings suggest that the tripartite interactions of CaMKII, GluN2B, and alpha-actinin-2 crosslink a membrane channel to the cytoskeleton beneath the synapse, and there are plenty of empty binding sites that other proteins can bind to. Moreover, the fact that certain interactions would generate autonomous enzymatic activity in CaMKII (Bayer et al., 2001; Saneyoshi et al., 2019) suggest a possible role in biochemical signalling for synaptic memory.

Recent evidence indicates that the CaMKII and GluN2B in synapses can undergo liquid–liquid phase separation, a process that involves biomolecules separating into distinct components within a cell (Hosokawa et al., 2021; Yasuda et al., 2022). Actin bundling can also be modulated by liquid–liquid phase separation in synapses (Chen et al., 2023), so it would be interesting to explore if this process has a role in changing and/or maintaining the structure of dendritic spines.
